# Supplementary material for: Mid-infrared emissivity of partially dehydrated asteroid (162173) Ryugu shows strong signs of aqueous alteration
Source: Nat Commun. 2022 Jan 18;13:364. doi: 10.1038/s41467-022-28051-y (PMC8766556; doi:10.1038/s41467-022-28051-y)
Supplement: Supplementary file 1 — Supplementary Information [file 41467_2022_28051_MOESM1_ESM.pdf]

Supplementary Information: Mid-Infrared Emissivity of Partially Dehydrated Asteroid (162173) Ryugu Shows Strong Signs of Aqueous Alteration

M. Hamm*[1,2], M. Grott[2], H. Senshu[3], J. Knollenberg[2], J. de Wiljes[1], V. E. Hamilton[4], F. Scholten[2], K. D. Matz[2], H. Bates[5,6], A. Maturilli[2], Y. Shimaki[7], N. Sakatani[8], W. Neumann[2,9], T. Okada[7], F. Preusker[2], S. Elgner[2], J. Helbert[2], E. Kührt[10,11], T.-M. Ho[12], S. Tanaka[7], R. Jaumann[13], S. Sugita[14]

1 – Institute of Mathematics, University of Potsdam, Potsdam, Germany

2 – Institute of Planetary Research, German Aerospace Center (DLR), Berlin, Germany

3 – Planetary Research and Exploration Center, Chiba Institute of Technology, Narashino, Japan

4 – Southwest Research Institute, Boulder, CO USA

5 – Department of Earth Sciences, Natural History Museum, Cromwell Road, London, SW7 5BD UK

6 – Atmospheric, Oceanic and Planetary Physics, Oxford University, Parks Road, Oxford, OX1 3PU, UK

7 – Institute of Space and Astronautical Science, Japan Aerospace Exploration Agency, Sagamihara, Japan

8 – Department of Physics, Rikkyo University, Toshima, Japan

9 – Klaus-Tschira-Labor für Kosmochemie, Institut für Geowissenschaften, Universität Heidelberg, Im Neuenheimer Feld 234-236, 69120 Heidelberg, Germany.

10 – Institute of Optical Sensor Systems, German Aerospace Center, Berlin, Germany

11 – Qian Xuesen Laboratory of Space Technology, China Academy of Space Technology, Beijing, China

12 – German Aerospace Center (DLR), Institute of Space Systems, Bremen, Germany

13 – Freie Universität Berlin, Berlin, Germany

14 – University of Tokyo, Tokyo, Japan

Corresponding Author: Maximilian Hamm, maximilian.hamm@dlr.de

Supplementary Note 1: Results obtained with the simplified thermal model

As described in the methods section, we used a simplified thermal model neglecting the sunlight reflection of MASCOT and the sub-facet surface roughness to estimate the systematic uncertainties that such model assumptions introduce to our analysis. This section provides a summary of the results obtained with the simplified model. Supplementary Fig. 1 shows the modelled brightness temperatures, based on the posterior parameter distributions in comparison to the observed brightness temperature in the six MARA filters. The afternoon and nighttime temperatures fit worse than in the main model and earlier temperatures are systematically underestimated and much better explained by the main model. Supplementary Fig. 2 shows the corresponding histograms of the posterior parameter combinations. Applying the basic model results in a higher thermal inertia estimate 298 ± 4 J m^{-2} K^{-1} s$^{-1/2}$ corresponding to a porosity of $42.8\ \%^{+0.3}_{-0.4}$. The broadband emissivity of the surface is $\varepsilon = 0.98^{+0.01}_{-0.01}$. These results of the narrow band emissivity estimates are similar to the main model basic model. Here the drop in the 5.5 – 7 µm band is less pronounced $\varepsilon_{B06} = 0.91^{+0.02}_{-0.02}$ whereas the emissivity in the 13.5 – 15.5 µm band is slightly lower $\varepsilon_{B13} = 0.95^{+0.01}_{-0.02}$. These differences, despite being outside the uncertainty given by the main model, do not change the interpretation of our results.

Supplementary Fig. 3 shows the comparison between the full model shown in the main article and previous works[1] for the W10 channel. The figure highlights the improvement around sunrise and also shows the similarity between the reduced model and previous works. The reduced model (Supplementary Fig. 1) is almost identical to the previous studies but with surface orientation and radiative heat exchange with the surrounding being fixed. Further, surface roughness is also included to some degree via the boulder DEM

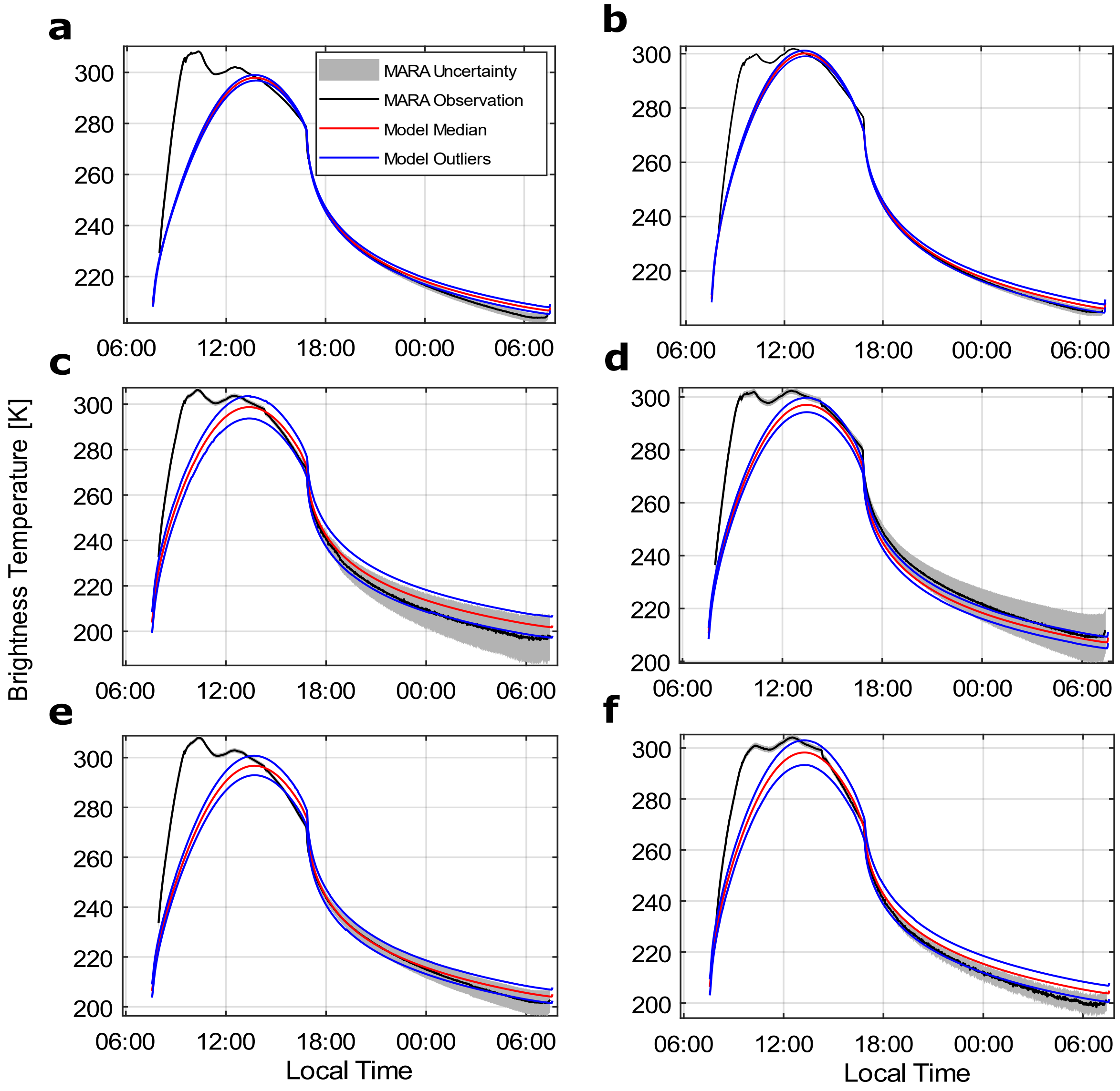

Supplementary Figure 1: Comparison of MARA observations to modelled observation based on a simplified model. a: Brightness temperature (black) observed in the Silicon-Longpass (SiLP) is shown as a function of local time (given in hours, defined by 1/24 of Ryugu's rotation period) along with the corresponding uncertainty (2σ with σ the standard deviation) given by grey shades. Red line indicates the median of modelled observation based on the posterior parameter estimation. Blue lines are models based on the range of outliers of the posterior within 1.5 times the interquartile range from the 25th and 75th percentile respectively. **b-f**: as for a but for the other instrument channels b: 8 – 12µm filter (W10), c: 5.5 – 7 µm (B06), d: 8 – 9.5 µm (B08), e: 9.5 – 11.5 µm (B09), and f: 13.5 – 15.5 µm (B13).

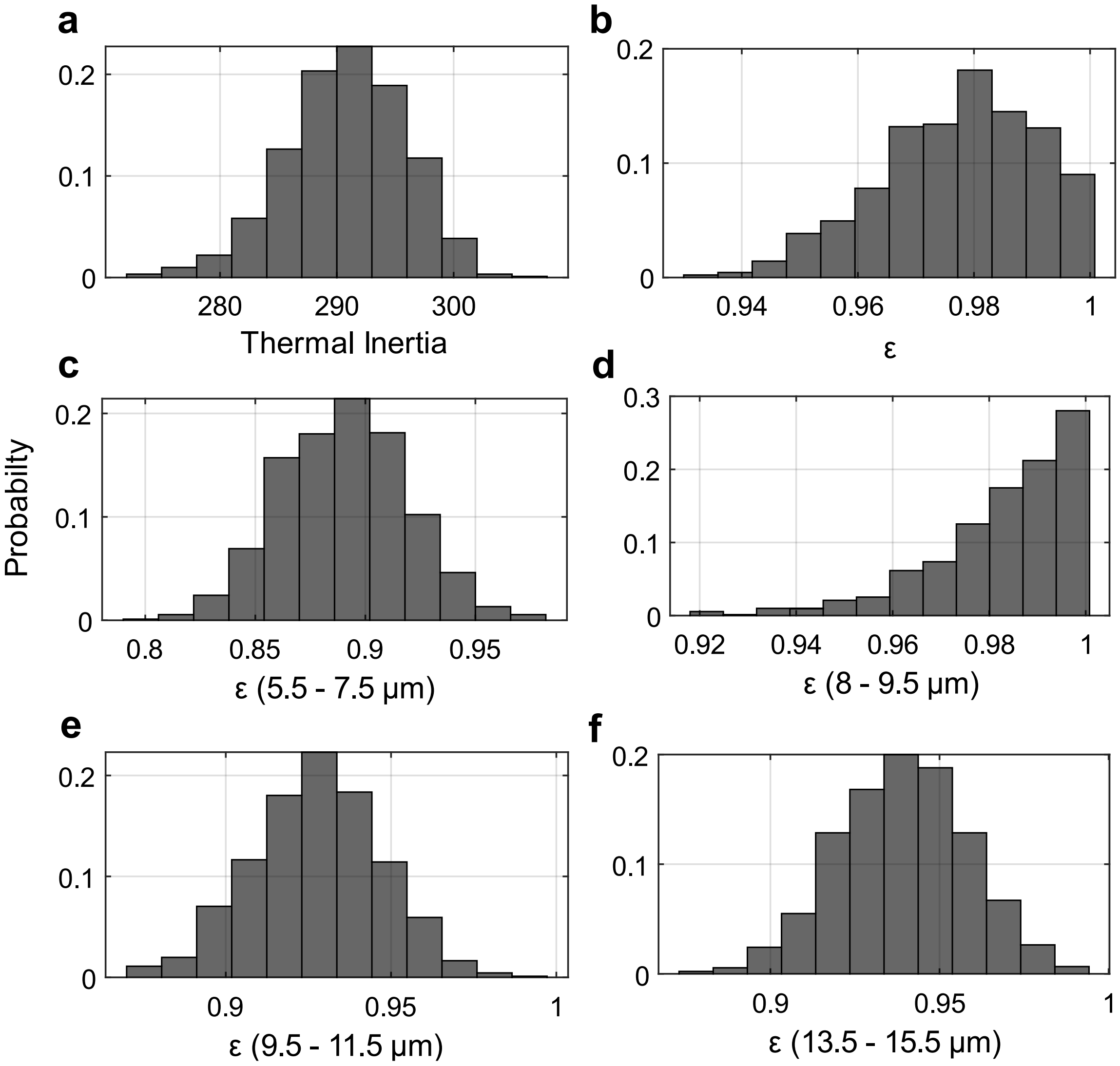

Supplementary Figure 2: Histograms of the posterior distribution of the six free model parameters of the simplified model. a thermal inertia (in units of $\mathrm{Jm^{-2}K^{-1}s^{-1/2}}$), **b** broadband emissivity, and the emissivity in the narrow bands: **c** B06, **d** B08, **e** B09, and **f** B13, with y-axis representing the probability of a parameter value within the interval given by the histogram bars. Parameter combinations with emissivity > 1 are discarded. The histograms are formed from the results of the data assimilation and represent the uncertainty of the parameter estimation according to the observation uncertainty. The estimated thermal inertia is slightly higher, the overall emissivity slightly higher compared to the main model. The estimated emissivity in the narrow filter bands are qualitatively similar.

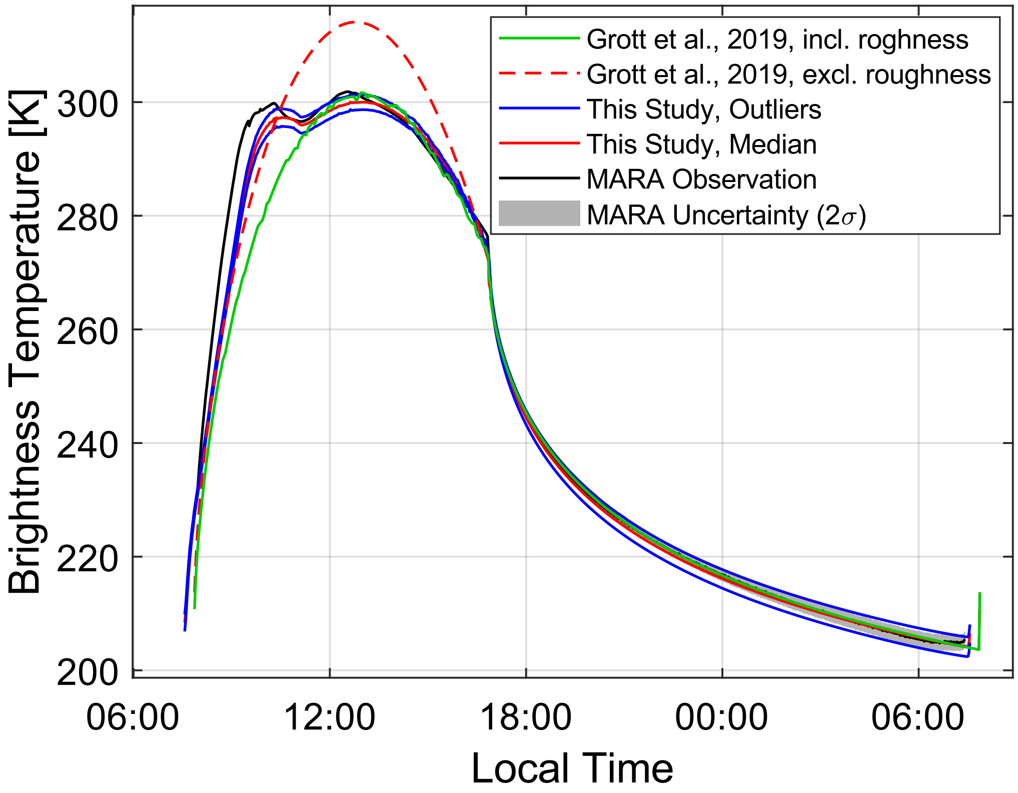

Supplementary Figure 3: Temperature prediction of the full model compared to earlier results. Observed brightness temperature (black) in the W10 band is shown as a function of local time (in hours, defined by 1/24 of Ryugu's rotation period) with grey indicating the 2σ uncertainty. The Solid red line represents the temperature prediction of the full thermal model based on the first, second, and third quartile of estimated model parameters, blue solid lines represent the temperature prediction based on the outliers of the parameter estimation. The results from Grott et al. (2019)[1] are shown for comparison, with the red dashed line representing the best-fitting model prediction without roughness and the green solid line representing the model prediction including roughness correction for the daytime temperatures. The presented full model explains the daytime temperatures compared to earlier simplified models better.

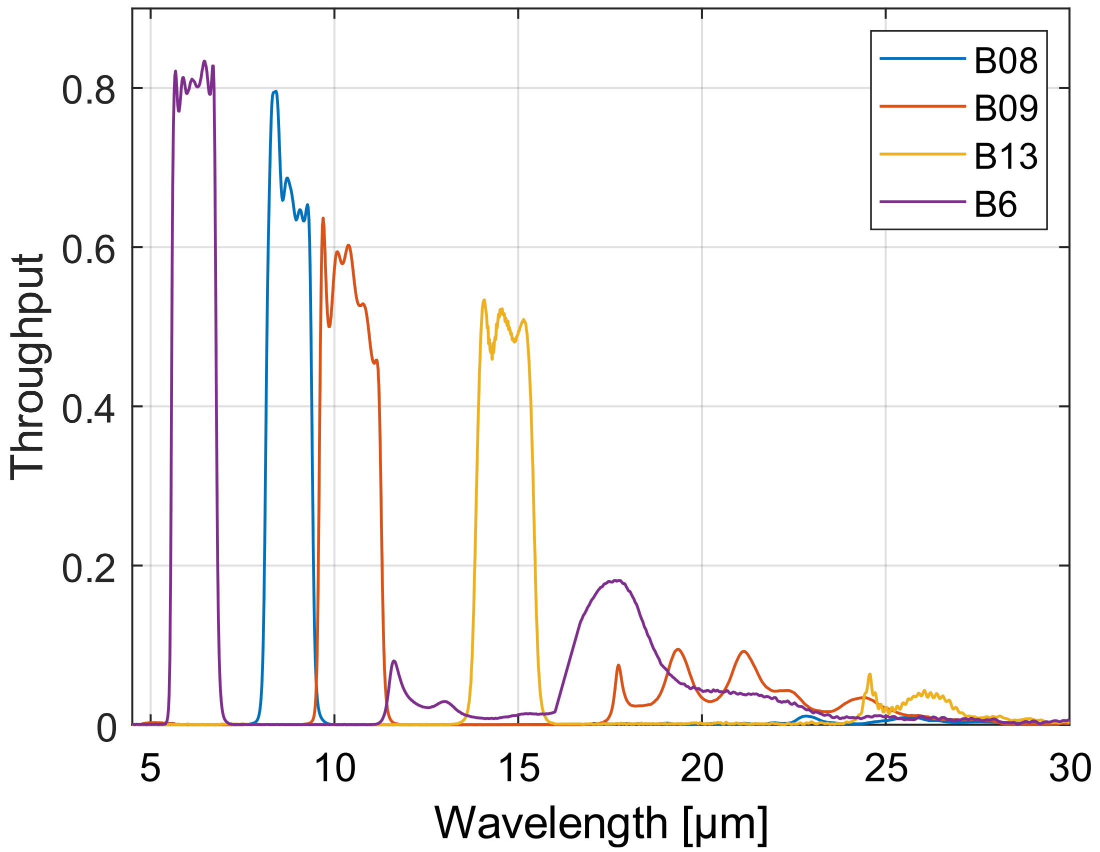

Supplementary Figure 4: Throughput of the four narrowband filter used for determining the emissivity variation within the mid-infrared wavelength region.[2] Throughput, combination of transmissivity of the filter and absorption of the sensor absorber, is plotted against wavelength. Secondary throughput windows appear at wavelength larger 15 µm and are most pronounced in the B06 filter

Chondrite Type	Meteorite Name
CI1	Orgueil P11442[3], Orgueil USNM 6765-2, Alais P10910
C1-ung	MIL 090292,13
CM1	Moapa Valley UA 2565[3], MIL 13037,3, EET 83334,12, EET 16100,5, MET 01070,40, MIL 13325,4, MIL 13063,3
CM1/2	ALH 83100,189[3], MAC 02820,3, MIL 13005,7, MIL 07677,5, EET 16129,5, MIL 090288,8, ALH 83100,172, MIL 11272,7, EET 90047,3, MIL 090992,6, MIL 090993,4, MCY 05231,15
CM2	LAP 02277,38[3], LAP 031166,16 [3], ALH 85013,13[3], Cold Bokkeveld UA 2409[3], Murray UA 2410 [3], Murchison UA 2406[3], QUE 93005,44, QUE 99355,29, MIL 07700,33, MET 00432,8, Nogoya UA 2407, Nogoya1, LEW 90500,60, ALH A81002,7, Murchison USNM-5487-1, Murchison USNM-5487-3, DNG 06004,24, Kivesvaara, Murray BM 1955, LAP 02336,5, LAP 03718,7, LAP 031214,6, LAP 031214,7, QUE 97990,53, LAP 02333,28, LAP 04514,6, DOM 08013,18
CM2 (heated)	WIS 91600,22, EET 96029,23, EET 87522,30, PCA 91008,6, PCA 02010,11, PCA 02012,1
CR1	GRO 95577,61, GRO 95577,76
CR2	LAP 04720,16, LAP 04516,1, LAP 02342,44, MET 00426,33, MIL 090001,38, GRA 06100,6
C2-ung	Essebi USNM-3200-5, Bells, EET 83355,15, MAC 87300,2
CO3	ALH A77307,87, Y-81020, Colony BM 1983, Kainsaz, Felix, Ornans, Lance, ALH 83108,12, Moss USNM-7319-5, Isna UA 2403
CO3 (anom.)	EET 90043,9
CV3	ALH 85006,10, Grosnaja, Mokoia NHM round, Leoville, Vigarano ASU 590, Allende matrix, C-17, Allende ASU 818.132-L1a
C3-ung	LEW 85332,11, MAC 88107,2
CK4	ALH 85002,8
CK5	EET 90026,8
CK6	MIL 13247,2, LEW 87009,11
CB	MIL 07588,4
ung.3	Acfer 094 USNM 7233

Supplementary Table 1: Overview of thin section chondrites data. The table lists the designation, type and source of the spectra of thin sections of chondrites that are shown in the main text's Fig. 4 and 5. The superscript refers to the referenced sources, the remainder of the data is previously published and was contributed by co-author V. E. Hamilton.

Chondrite Type	Meteorite Name
CI1	Orgueil[4], Ivuna[4]
CM1	GRO 95645[4], NWA 4765[4], LAP 02277[4], Moapa Valley[4], MIL 05137[4], MIL 07689[4], MET 01070[5]
CM1/2	MCY 05231[4], LAP 031214[4], MIL 090288[4], LAP 031166[4], NWA 8534[4], ALH 83100[6]
CM2	Murchison[4], MAC 88100[5], QUE 97990[5], MCY 05230[5], LEW 87022[5], MIL 07700[5], LAP 02336[5], ALH 84033[5], ALH 84044[5], DOM 08003[5], EET 87522[5], LAP 03718[5], LON 94101[6], ALH 83102[6]
CR1	GRO 95577[5]
CR2	GRO 03116[5], EET 92159[5], GRA 06100[5], MIL 090001[7]
CM2 (heated)	PCA 02010[6], PCA 02012[6], EET 96029[6], Y 793321[6], Y 86695*, Y 82054*, Y 82098*, A 881458*
CI1 (heated)/CY	Y 980115[6]
C2 – ung. (heated)/ CY	B 7904[6], Y 86720[6], Y 86789[6]
CM (anom.)	LEW 85312[5], LEW 85311[5], WIS 91600[6]
CV 3	Allende[8]

Supplementary Table 2: Overview of powdered chondrites data. The table lists the designation, type and source of the powdered spectra of chondrites that are shown in the main text's Fig. 6 and 7. The superscript refers to the referenced sources, asterisk indicate previously unpublished data by co-author A. Maturilli.

Supplementary References

1. Grott, M. *et al.* Low thermal conductivity boulder with high porosity identified on C-type asteroid (162173) Ryugu. *Nat Astron* **3**, 971–976 (2019).
2. Grott, M. *et al.* The MASCOT Radiometer MARA for the Hayabusa 2 Mission. *Space Sci Rev* **208**, 413–431 (2017).
3. Hamilton, V. E. *et al.* Evidence for limited compositional and particle size variation on asteroid (101955) Bennu from thermal infrared spectroscopy. *A&A* **650**, A120 (2021).
4. Bates, H. C., King, A. J., Hanna, K. L. D., Bowles, N. E. & Russell, S. S. Linking mineralogy and spectroscopy of highly aqueously altered CM and CI carbonaceous chondrites in preparation for primitive asteroid sample return. *Meteoritics & Planetary Science* **55**, 77–101 (2020).
5. Beck, P. *et al.* What is controlling the reflectance spectra (0.35–150 µm) of hydrated (and dehydrated) carbonaceous chondrites? *Icarus* **313**, 124–138 (2018).
6. Bates, H. C., Donaldson Hanna, K. L., King, A. J., Bowles, N. E. & Russell, S. S. A Spectral Investigation of Aqueously and Thermally Altered CM, CM-An, and CY Chondrites Under Simulated Asteroid Conditions for Comparison With OSIRIS-REx and Hayabusa2 Observations. *Journal of Geophysical Research: Planets* **126**, e2021JE006827 (2021).
7. Donaldson Hanna, K. L. *et al.* Spectral Characterization of Bennu Analogs Using PASCALE: A New Experimental Set-Up for Simulating the Near-Surface Conditions of Airless Bodies. *J Geophys Res Planets* **126**, e2020JE006624 (2021)
8. Maturilli, A., Helbert, J., Ferrari, S., Davidsson, B. & D'Amore, M. Characterization of asteroid analogues by means of emission and reflectance spectroscopy in the 1- to 100-µm spectral range. *Earth Planet Sp* **68**, 113 (2016).
